# The Synergistic Roles of Cholecystokinin B and Dopamine D_5_ Receptors on the Regulation of Renal Sodium Excretion

**DOI:** 10.1371/journal.pone.0146641

**Published:** 2016-01-11

**Authors:** Xiaoliang Jiang, Wei Chen, Xing Liu, Zihao Wang, Yunpeng Liu, Robin A. Felder, John J. Gildea, Pedro A. Jose, Chuan Qin, Zhiwei Yang

**Affiliations:** 1 Institute of Laboratory Animal Science, Chinese Academy of Medical Sciences (CAMS) & Comparative Medicine Centre, Peking Union Medical Collage (PUMC), Beijing, P. R. China; 2 Department of Pathology, University of Virginia School of Medicine, Charlottesville, Virginia, United States of America; 3 Division of Nephrology, Departments of Medicine and Physiology, University of Maryland School of Medicine, Baltimore, Maryland, United States of America; 4 CollaborativeInnovation Center for Cardiovascular Disorders, Beijing, P. R. China; University of Geneva, SWITZERLAND

## Abstract

Renal dopamine D_1_-like receptors (D_1_R and D_5_R) and the gastrin receptor (CCK_B_R) are involved in the maintenance of sodium homeostasis. The D_1_R has been found to interact synergistically with CCK_B_R in renal proximal tubule (RPT) cells to promote natriuresis and diuresis. D_5_R, which has a higher affinity for dopamine than D_1_R, has some constitutive activity. Hence, we sought to investigate the interaction between D_5_R and CCK_B_R in the regulation of renal sodium excretion. In present study, we found D_5_R and CCK_B_R increase each other’s expression in a concentration- and time-dependent manner in the HK-2 cell, the specificity of which was verified in HEK293 cells heterologously expressing both human D_5_R and CCK_B_R and in RPT cells from a male normotensive human. The specificity of D_5_R in the D_5_R and CCK_B_R interaction was verified further using a selective D_5_R antagonist, LE-PM436. Also, D_5_R and CCK_B_R colocalize and co-immunoprecipitate in BALB/c mouse RPTs and human RPT cells. CCK_B_R protein expression in plasma membrane-enriched fractions of renal cortex (PMFs) is greater in D_5_R^-/-^ mice than D_5_R^+/+^ littermates and D_5_R protein expression in PMFs is also greater in CCK_B_R^-/-^ mice than CCK_B_R^+/+^ littermates. High salt diet, relative to normal salt diet, increased the expression of CCK_B_R and D_5_R proteins in PMFs. Disruption of CCK_B_R in mice caused hypertension and decreased sodium excretion. The natriuresis in salt-loaded BALB/c mice was decreased by YF476, a CCK_B_R antagonist and Sch23390, a D_1_R/D_5_R antagonist. Furthermore, the natriuresis caused by gastrin was blocked by Sch23390 while the natriuresis caused by fenoldopam, a D_1_R/D_5_R agonist, was blocked by YF476. Taken together, our findings indicate that CCK_B_R and D_5_R synergistically interact in the kidney, which may contribute to the maintenance of normal sodium balance following an increase in sodium intake.

## Introduction

Hypertension occurs as a consequence of a complex interplay among multiple genetic, epigenetic, and environmental determinants [1]. Salt consumption is an important non-genetic determinant, and excessive dietary salt intake can increase blood pressure in genetically susceptible individuals [2]. Recent population-based studies have revealed a nonlinear with even a J-shaped correlation between salt intake and blood pressure or cardiovascular disease mortality [3–5]. An increasing number of hormones, via their receptors, have been reported to regulate ion exchangers, transporters, channels, and pumps in renal tubules, including the renal proximal tubule (RPT), that are crucial in maintaining normal sodium balance [6,7].

Dopamine, secreted in the kidney mainly by RPT cells, via its receptors that are classified into “D_1_-like” (D_1_R and D_5_R) and “D_2_-like” (D_2_R, D_3_R and D_4_R) receptors, is responsible for over 50% of renal sodium excretion during conditions of mild volume and sodium excess [8–10]. The acute infusion of fenoldopam, a D_1_-like receptor agonist, induces natriuresis and diuresis in humans, rats, and mice [8–14]. Disruption of any of the dopamine receptor gene subtypes in mice causes hypertension which can be aggravated by salt loading that is dopamine receptor subtype dependent [10].

Gastrointestinal hormones have been reported to be involved in the regulation of renal sodium excretion and blood pressure [14,15]. An oral sodium load causes a greater natriuresis than an intravenous infusion of the same amount of sodium [16–18], suggesting that gastrointestinal hormones have a role in regulating the postprandial natriuretic response. One such hormone may be gastrin. Mice lacking the gastrin gene are hypertensive and salt-sensitive [18]. The receptor of gastrin, CCK_B_R, has been reported to be expressed in several nephron segments, including the RPT and collecting duct [18–21]. Gastrin, which is taken up by RPT to a greater extent than other gut hormones [22], via CCK_B_R, can induce natriuresis and diuresis by inhibiting the activities of renal Na^+^-K^+^-ATPase and sodium/hydrogen exchanger type 3 (NHE3) [14,18–20].

Our recent study reported a synergistic interaction between gastrin, via CCK_B_R, and D_1_R, one of the two D_1_-like receptors, in promoting water and sodium excretion [14]. The other D_1_-like receptor, D_5_R, has a 30% homology in the N and C termini and an 80% homology in the transmembrane domain with the D_1_R. D_5_R and D_1_R, via a D_1_R/D_5_R heteromer, cooperatively decrease sodium transport in RPT cells by inhibition of NHE3 and Na^+^-K^+^-ATPase activities [23]. The D_5_R may be more important than D_1_R in regulating salt balance because D_5_R has some constitutive activity and a higher affinity for dopamine than D_1_R [24,25]. Therefore, in this study, we tested the hypothesis that D_5_R and CCK_B_R synergistically regulate each other in the kidney, specifically in RPT cells, which may have important implications in the regulation of renal sodium excretion.

## Materials and Methods

### Materials

We used immortalized human RPT cells (HK-2) (China Center for Type Culture Collection, 3115CNCB00336, Wuhan, China), as well as well-characterized human embryonic kidney 293 (HEK293) cells, heterologously expressing human D_5_R [26,27], and RPT cells from a normotensive Caucasian male (NT) [28]. Adult (4-month old) male BALB/c mice were bought from Beijing HFK Bioscience Co, LTD. Sixth generation progeny D_5_R^-/-^ and CCK_B_R^-/-^ mice were obtained from Jackson Laboratory and bred in an AAALAC-accredited facility. The 4-month male D_5_R^-/-^ and CCK_B_R^-/-^ mice and their littermates were used for further study. All animal-related studies were approved by the Institutional Animal Care and Use Committee of the Institute of Laboratory Animal Science, Peking Union Medical Collage, China. The information of all the chemical drugs, antibodies, and related test kits are listed in S1 Table.

### Cell culture

All the cells (HK-2, HEK293, and NT), in DMEM/F12 with 4.5 g/L D-glucose, supplemented with 10% fetal bovine serum, 100 μg/ml penicillin and 10 μg/ml streptomycin, were cultured in a humidified cell culture incubator maintained at 37°C and supplied with 5% CO_2_ and 95% O_2_. We used cells with low passage numbers (<20 for HK-2 and NT cells and <40 for HEK293 cells) to avoid the confounding effects of cellular senescence. The cells tested negative for mycoplasma infection.

### Co-transfection

HEK293 cells stably overexpressing full-length human D_5_R with blasticidin resistance (HEK293-D_5_R) were previously generated in our laboratory [26,27]. HEK293-D_5_R cells were transfected with pCMV6-AC vector with full-length human CCK_B_R cDNA, using Lipofectamine 2000 transfection reagents, according to the manufacturer’s protocol. Stably transfected single colonies, selected with 600 μg/ml neomycin for the CCK_B_R-positive colonies, were designated as HEK293-D_5_R-CCK_B_R cells.

### Immunoblotting

Whole cell lysates were extracted in ice-cold RIPA lysis buffer, sonicated, kept on ice for 30 minutes, and centrifuged with 14000 g for 30 minutes at 4°C. PMFs were extracted using a membrane protein extraction Kit (Sango Biotech, China), in accordance with the manufacturer’s instruction. Detailed steps are shown in S1 File. Protein concentration was determined by bicinchoninic acid assay using bovine serum albumin as a standard. Equal amounts of protein (60 μg for whole cell lysates and 40 μg for PMFs) were subjected to immunoblotting. The densitometry values of whole cell lysates were normalized by the expression of GAPDH. The primary antibodies are mouse polyclonal anti-D_5_R (Santa Cruz, USA) and rabbit polyclonal anti-CCK_B_R (NOVUS, USA) whose specificities have been reported [29,30].

### Quantitative Real-Time PCR

Total mRNA was purified using 1ml Trizol and quantified using a spectrophotometer. The RNA samples were reverse-transcribed using SuperScript III. Gene expression was quantified by real-time PCR, using an Applied Biosystem 7500 Real-Time PCR System. The assay used gene specific primers and One Step SYBR PrimeScript RT-PCR Kit, as described in the manufacturer’s manual. All the primers used in this study are provided in S2 Table. Data were analyzed using the △△Ct method [31].

### Co-immunoprecipitation

Co-immunoprecipitation was performed using an immunoprecipitation kit. Equal amounts of whole cell lysates (500 μg protein) were mixed with mouse anti-D_5_R antibody (Santa Cruz, USA), non-immune mouse serum (negative control), or mouse anti-CCK_B_R antibody (positive control, Santa Cruz, USA) whose specificity has been reported [32]. Protein A/G agarose beads were added and incubated overnight at 4°C. The bound proteins were eluted using 30 μl of Laemmli buffer. The samples were subjected to immunoblotting and probed for CCK_B_R using a rabbit anti-CCK_B_R antibody (Novus, USA). Reverse co-immunoprecipitation was performed using the same method; cell lysates were mixed with mouse anti-CCK_B_R antibody (Santa Cruz, USA), non-immune mouse serum (negative control), or mouse anti-D_5_R (positive control, Santa Cruz, USA) and the bound proteins were subjected to immunoblotting and probed for D_5_R, using a rabbit anti-D_5_R antibody (Santa Cruz, USA) whose specificity has been reported [33,34].

### Confocal microscopy of double-strained HK-2 cells and RPTs of BALB/c mouse

HK-2 cells, grown on coverslips, were fixed with ice-cold methanol for 30 minutes. Five-micron sections were cut from formalin-fixed and paraffin-embedded BALB/c mouse kidneys. CCK_B_R was visualized using a polyclonal rabbit anti-CCK_B_R antibody (NOVUS, USA), followed by Alexa Fluor 568-labeled goat anti-rabbit secondary antibody (Abcam, USA). D_5_R was visualized using a polyclonal mouse anti-D_5_R antibody (Santa Cruz, USA), followed by Alexa Fluor 488-labeled goat anti-mouse secondary antibody (Abcam, USA). For a negative control, the primary antibodies were substituted with non-immune rabbit or mouse serum at an appropriate dilution. Colocalization of the D_5_R and CCK_B_R was identified by the development of a yellow color in the merged images.

### Blood pressure measurement

Blood pressure was measured from the aorta, via the left carotid artery, under pentobarbital (60 mg/kg, administered intraperitoneally) anesthesia. Subsequently, the mice were sacrificed by neck dislocation; the kidneys were harvested and samples were prepared for immunoblotting.

### Sodium excretion detection

BALB/c mice, CCK_B_R^-/-^ mice and CCK_B_R^+/+^ littermates were acclimatized in metabolic cages for 3 days, then divided into two groups and fed normal (**0.4% NaCl**) or high-salt (**3% NaCl**) diet for two weeks. Afterwards, BALB/c mice on high salt diet were separated into seven groups and intraperitoneally injected with vehicle (normal saline, 0.5 ml), Sch23390 (a D_1_-like receptor antagonist, 0.1 mg/kg) [14,27,33,35], YF476 (a CCK_B_R antagonist, 0.1 mg/kg) [36,37], fenoldopam (a D_1_-like receptor agonist, 1mg/kg) [8–14], gastrin (a CCK_B_R ligand, 10 μg/kg) [14,18–20], fenoldopam (1mg/kg) coupled with YF476 (0.1 mg/kg) and gastrin (10 μg/kg) coupled with Sch23390 (0.1 mg/kg), respectively, daily for one week. The BALB/c mice on normal salt diet were also injected intraperitoneally with 0.5 ml normal saline, daily for one week. At the end of drug treatment, urine was collected for 24 hours. Urine sodium concentration was measured using a Synchron EL-ISE Electrolyte system (Beckman, USA). Urine creatinine concentration was measured by an automated enzymatic method [38]. Thereafter, the mice were sacrificed by neck dislocation and the kidneys were harvested and frozen in liquid nitrogen until use.

### Statistics

The data are expressed as mean ± SEM. Significant difference between two groups was determined by Student’s *t*-test and one-way factorial ANOVA followed by Duncan’s multiple range test for groups>2. P<0.05 was considered significant.

## Results

### D_5_R and CCK_B_R co-regulation in HK-2 cells

**Fig 1:**
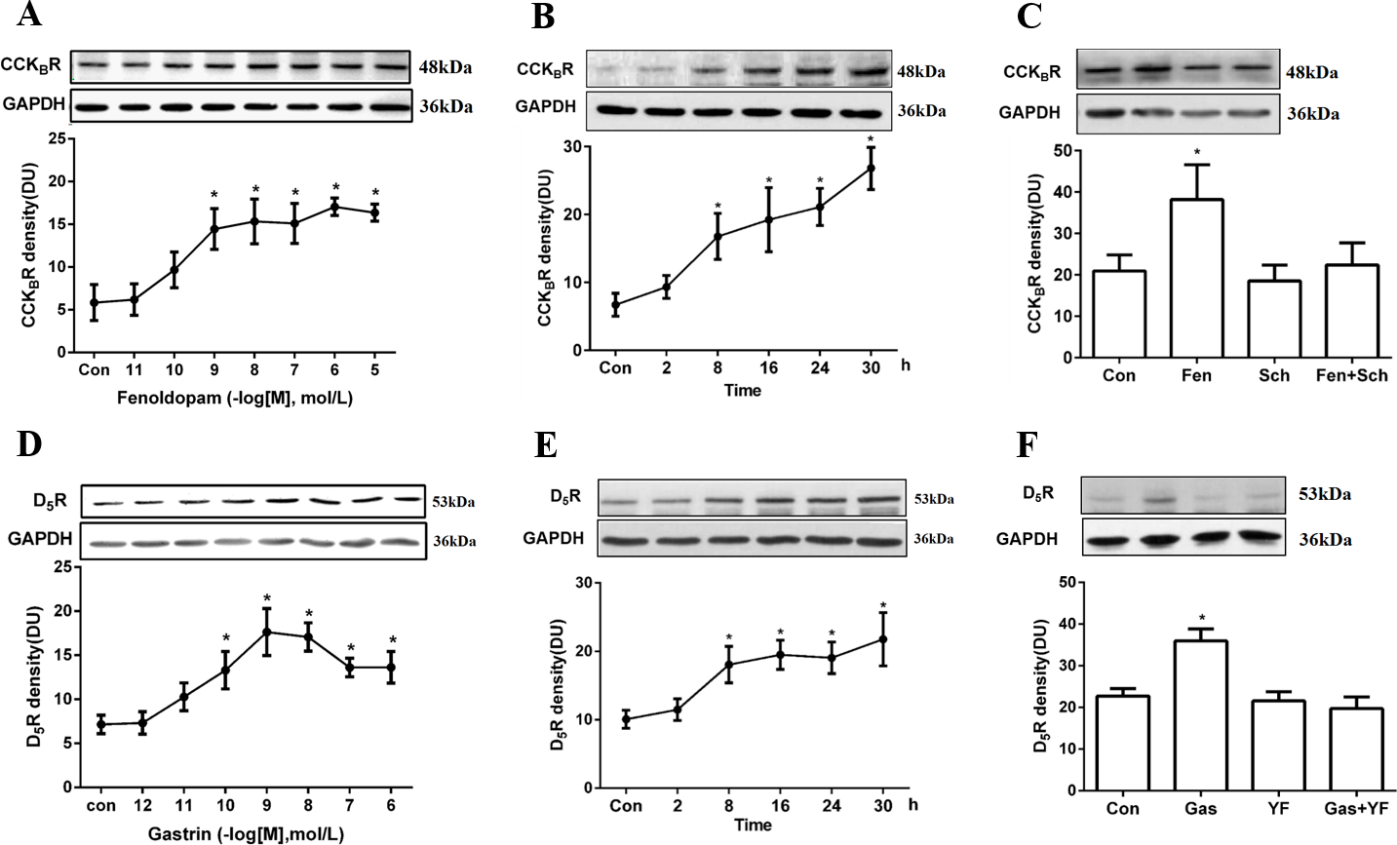
D_5_R and CCK_B_R co-regulation in HK-2 cells. (A) CCK_B_R protein expression in response to varying concentrations of the D_1_R and D_5_R agonist fenoldopam (10^−11^–10^−5^ mol/L, 24 hours, n = 6, *P<0.05 vs control, one-way factorial ANOVA, Duncan’s test). (B) CCK_B_R protein expression in response to varying durations of incubation with fenoldopam (0–30 hours, 10^−6^ mol/L, n = 6, *P<0.05 vs control, one-way factorial ANOVA, Duncan’s test). (C) Effects of fenoldopam (10^−6^ mol/L, 24 hours) and D_1_R and D_5_R antagonist Sch23390 (10^−6^ mol/L, 24 hours) on CCK_B_R protein expression (n = 5, *P<0.05 vs control, one-way factorial ANOVA, Duncan’s test). (D) D_5_R protein expression in response to varying concentrations of gastrin (10^−12^–10^−6^ mol/L, 24 hours, n = 6, *P<0.05 vs control, one-way factorial ANOVA, Duncan’s test). (E) D_5_R protein expression in response to varying durations of incubation with gastrin (0–30 hours, 10^−8^ mol/L, n = 6, *P<0.05 vs control, one-way factorial ANOVA, Duncan’s test). (F) Effects of gastrin (10^-8^mol/L, 24 hours) and CCK_B_R antagonist YF476 (10^−8^ mol/L, 24 hours) on D_5_R protein expression (n = 5, *P<0.05 vs control, one-way factorial ANOVA, Duncan’s test). All immunoblotting results are expressed as relative density units (DU) and normalized by GAPDH expression. Immunoblots of D_5_R, CCK_B_R, and GAPDH are shown in the inset.

In HK-2 cells, fenoldopam, a D_1_R and D_5_R agonist, increased CCK_B_R protein expression in a concentration- and time-dependent manner. The ability of fenoldopam (24 hours) to increase CCK_B_R protein was significant at ≥10^−9^ with a concentration for half-maximal stimulation (EC_50_) of 1.39 x 10^−10^ M (Fig 1A). The stimulatory effect of fenoldopam (10^−6^ M) was evident as early as 8 hours and lasted for at least 30 hours (Fig 1B). We next verified the specificity of the D_1_-like receptor stimulatory effect of fenoldopam (10^−6^ M, 24 hours) on CCK_B_R expression by using Sch23390, a D_1_-like receptor antagonist. As shown in Fig 1C, CCK_B_R protein expression was significantly increased with fenoldopam treatment, the effect of which was blocked by pre-incubation with Sch23390 (10^−6^ M, 24 hours), which by itself had no effect on CCK_B_R protein expression.

Gastrin, a CCK_B_R agonist, also increased D_5_R protein expression in a concentration- and time-dependent manner. The ability of gastrin to increase D_5_R protein was significant at ≥10^−10^ M with an EC_50_ of 1.76 x 10^−11^ M (Fig 1D). The stimulatory effect of gastrin (10^−8^ M) was evident as early as 8 hours and lasted for at least 30 hours (Fig 1E). The specificity of the stimulatory effect of gastrin (10^−8^ M, 24 hours) was determined by using CCK_B_R antagonist YF476 (10^−8^ M, 24 hours). The stimulatory effect of gastrin on D_5_R expression was abrogated by YF476, which by itself had no effect on D_5_R expression (Fig 1F).

### D_5_R and CCK_B_R co-regulation in HEK293-D_5_R-CCK_B_R cells

**Fig 2:**
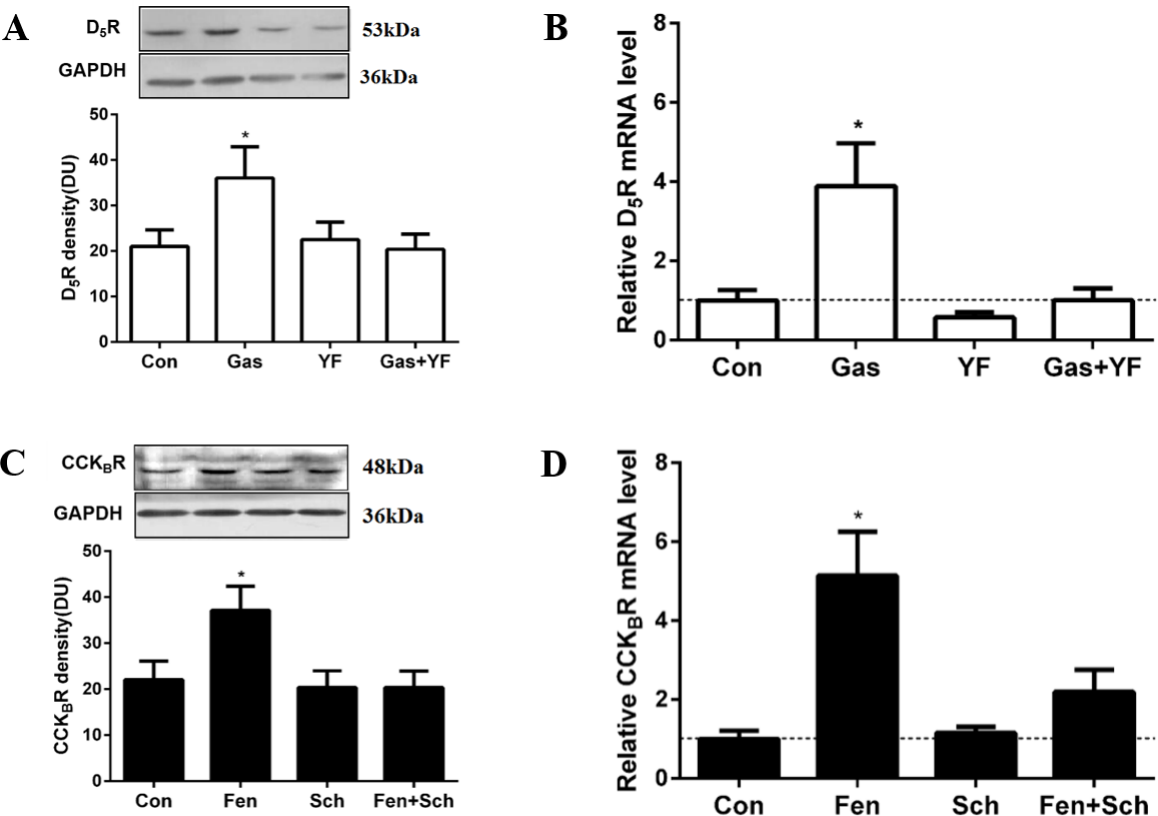
D_5_R and CCK_B_R co-regulation in HEK293-D_5_R-CCK_B_R cells. (A and B) Effects of gastrin (Gas, 10^−8^ mol/L, 3 hours) and CCK_B_R antagonist YF476 (YF, 10^−8^ mol/L, 3 hours) on D_5_R protein and mRNA expressions in HEK293-D_5_R-CCK_B_R cells (n = 6 for protein, n = 5 for mRNA, *P<0.05 versus others, one-way factorial ANOVA, Duncan’s test). (C and D) Effects of D_1_R and D_5_R agonist fenoldopam (Fen, 10^−6^ mol/L, 3 hours) and D_1_R and D_5_R antagonist Sch23390 (Sch, 10^−6^ mol/L, 3 hours) on CCK_B_R protein and mRNA expressions in HEK293-D_5_R-CCK_B_R cells (n = 6 for protein, n = 5 for mRNA, *P<0.05 versus others, one-way factorial ANOVA, Duncan’s test). Immunoblotting results are expressed as relative density units (DU). Immunoblots of D_5_R, CCK_B_R and GAPDH are shown in the inset. mRNA expression was determined by qRT-PCR and corrected for the expression of GAPDH mRNA.

Fenoldopam cannot distinguish the 2 subtypes of D_1_-like receptors, D_1_R and D_5_R, from each other [8–10, 12–14]. HEK293 cells express no endogenous D_1_R and some D_5_R [39]. Therefore, HEK293-D_5_R-CCK_B_R cells were generated to avoid the confounding effect of D_1_R. PCR and immunoblotting studies demonstrated stable HEK293-D_5_R-CCK_B_R cells over-expressing human D_5_R and CCK_B_R (S1 Fig). In these cells, gastrin increased D_5_R protein (1.7±0.2-fold, P<0.05) and mRNA (3.9±0.2-fold, P<0.05) expressions that were blocked by YF476, a CCK_B_R antagonist, which by itself had no effect (Fig 2A and 2B). Fenoldopam also significantly increased CCK_B_R protein (1.7±0.1 fold, P<0.05) and mRNA (5.1±0.3 fold, P<0.05) expressions, that were blocked by Sch23390, a specific D_5_R antagonist in the absence of D_1_R (Fig 2C and 2D). The results of the studies using HEK293-D_5_R-CCK_B_R cells combined with the results in HK-2 cells suggest a specific CCK_B_R-D_5_R interaction, independent of D_1_R, at both the transcriptional and translational levels.

### Direct and/or indirect interaction between D_5_R and CCK_B_R

**Fig 3:**
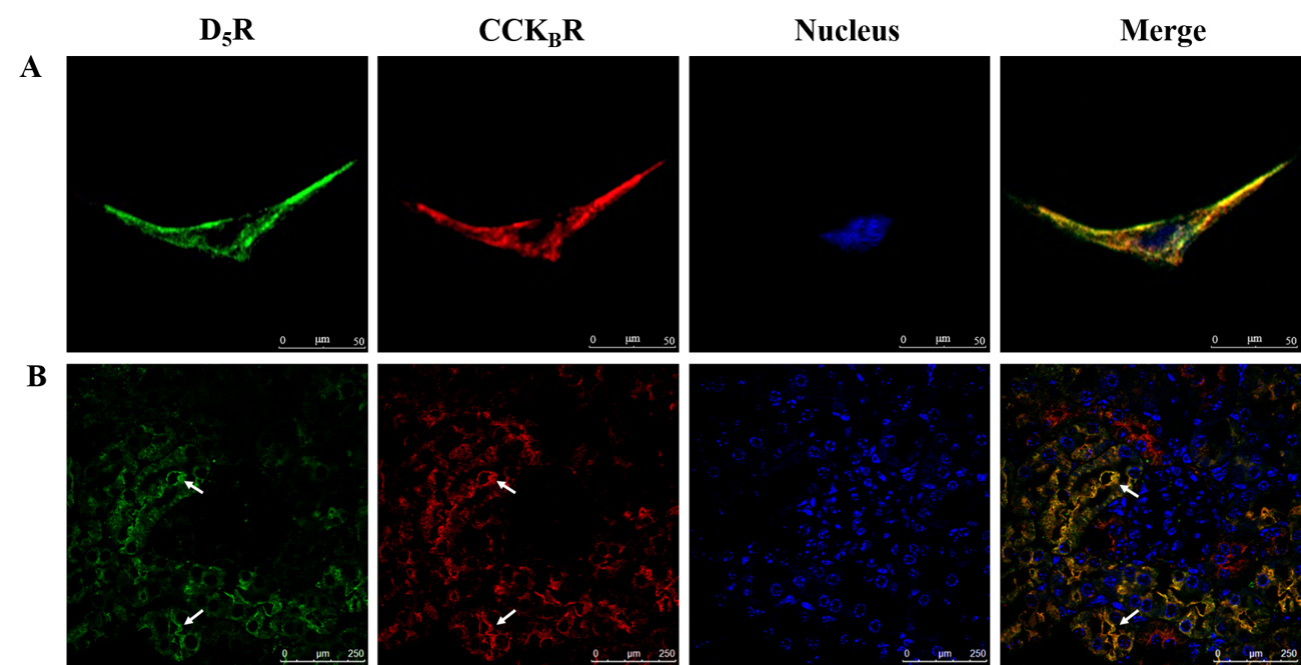
D_5_R and CCK_B_R colocalization in RPTs of BALB/c mice. Colocalization of Alexa Fluor 488-labeled D_5_R (green) and Alexa Fluor 568-labeled CCK_B_R (red) in (A) HK-2 cells and (B) the RPTs of BALB/c mice. The colocalization of D_5_R and CCK_B_R is illustrated by the yellow color in the merge images. Scale bar: 50 μm for HK-2 cells and 250 μm for the RPTs of BALB/c mice. The white arrows are pointing to the RPTs.

**Fig 4:**
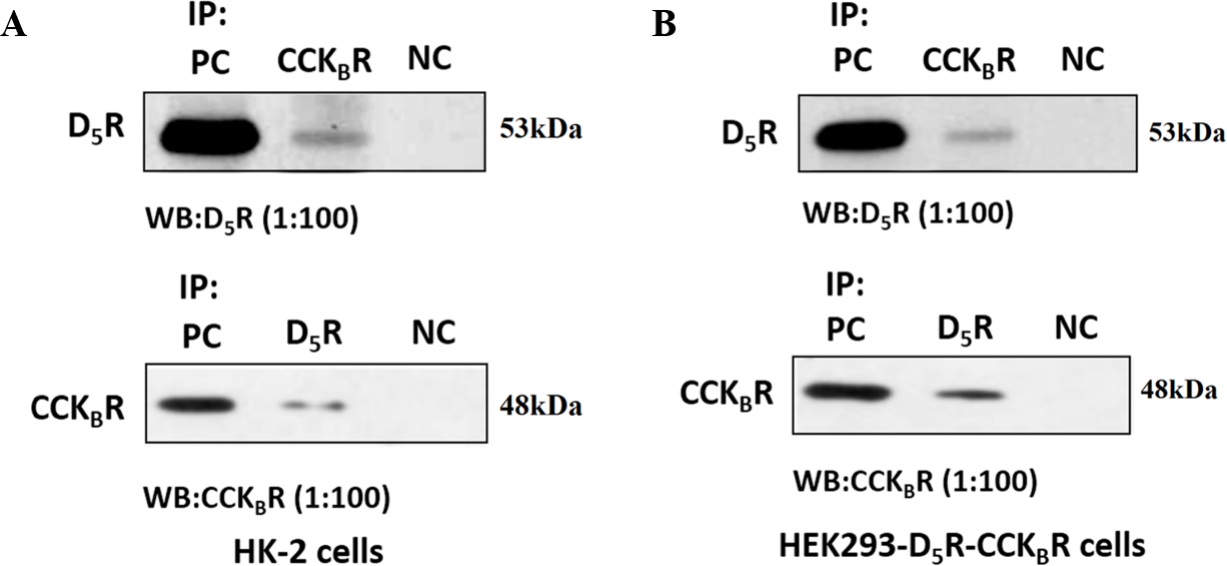
D_5_R and CCK_B_R physical interaction in HK-2 and HEK293-D_5_R-CCK_B_R cells. Co-immunoprecipitation of D_5_R and CCK_B_R in HK-2 cells (A) and HEK293-D_5_R-CCK_B_R cells (B). Whole cell lysates were subjected to immunoprecipitation (IP) with mouse anti-D_5_R antibody, mouse anti-CCK_B_R antibody, or non-immune mouse serum (negative control). Immunoprecipitated complexes were analyzed by immunoblotting (western blot, WB), using rabbit anti-D_5_R antibody or rabbit anti-CCK_B_R antibody. PC: positive control; NC: negative control. These experiments were repeated three times with similar results.

In order to affirm the potential for a direct or indirect interaction between D_5_R and CCK_B_R, we studied the co-localization of D_5_R and CCK_B_R in HK-2 cells and RPTs of BALB/c mice. Immunofluorescent staining showed that both D_5_R and CCK_B_R were mainly expressed and colocalized at the cell surface membranes of HK-2 cells (Fig 3A) and RPTs of BALB/c mice (Fig 3B**)**. We also performed a co-immunoprecipitation study to determine whether there is a physical interaction between D_5_R and CCK_B_R and found that D_5_R co-immunoprecipitated with CCK_B_R in both HK-2 (Fig 4A**)** and HEK293-D_5_R-CCK_B_R cells (Fig 4B). These data indicate that CCK_B_R and D_5_R can interact with each other via a direct and/or indirect way.

### Blood pressures in CCK_B_R^-/-^ mice and CCK_B_R^+/+^ littermates

**Fig 5:**
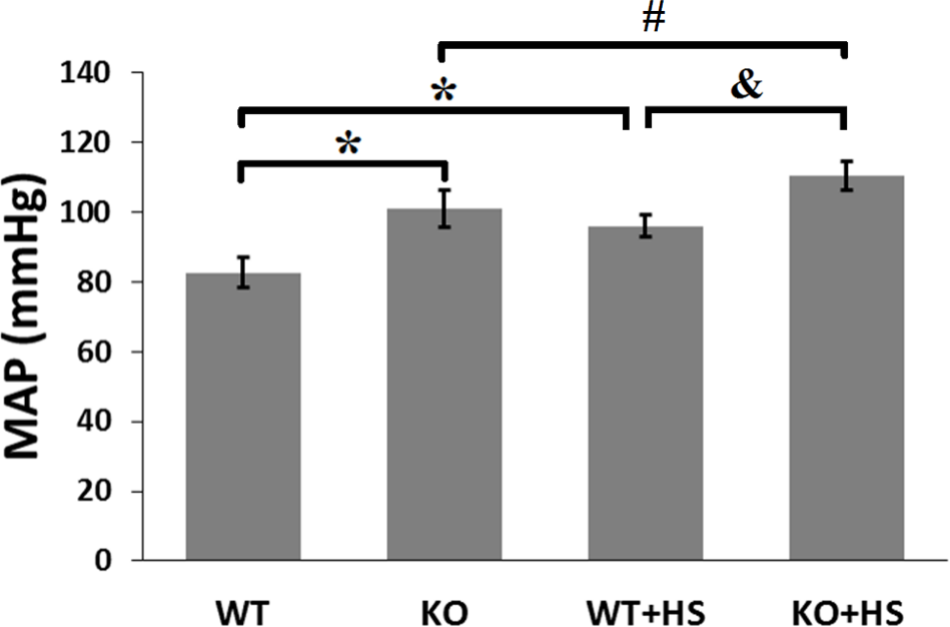
MAPs in CCK_B_R^-/-^ mice and CCK_B_R^+/+^ littermates. MAPs were measured from the aorta, via the left carotid artery, under pentobarbital anesthesia. WT and KO indicate CCK_B_R^+/+^ littermates (n = 19) and CCK_B_R^-/-^ mice (n = 21) on normal salt diet, respectively; WT+HS and KO+HS indicate CCK_B_R^+/+^ littermates (n = 11) and CCK_B_R^-/-^ mice (n = 8) on high salt diet, respectively. *P<0.05 vs WT, #P<0.05 vs KO, &P<0.05 vs WT+HS, one-way factorial ANOVA.

Mean arterial pressures (MAPs, Fig 5) measured under anesthesia were significantly higher in CCK_B_R^-/-^ mice than that in CCK_B_R^+/+^ littermates (101.0±5.3 mmHg vs 82.5±4.3 mmHg) on normal salt diet. Even though high salt diet further increased the MAPs of both CCK_B_R^-/-^ mice (110.4±4.0 mmHg vs 101.0±5.3 mmHg) and the corresponding littermates (96.0±3.3 mmHg vs 82.5±4.3 mmHg) compared with normal salt diet. High salt diet increased the MAPs of CCK_B_R^-/-^ mice than those of CCK_B_R^+/+^ littermates (110.4±4.0 mmHg vs 96.0±3.3 mmHg).

### D_5_R and CCK_B_R interaction and their expression in mouse kidney

**Fig 6:**
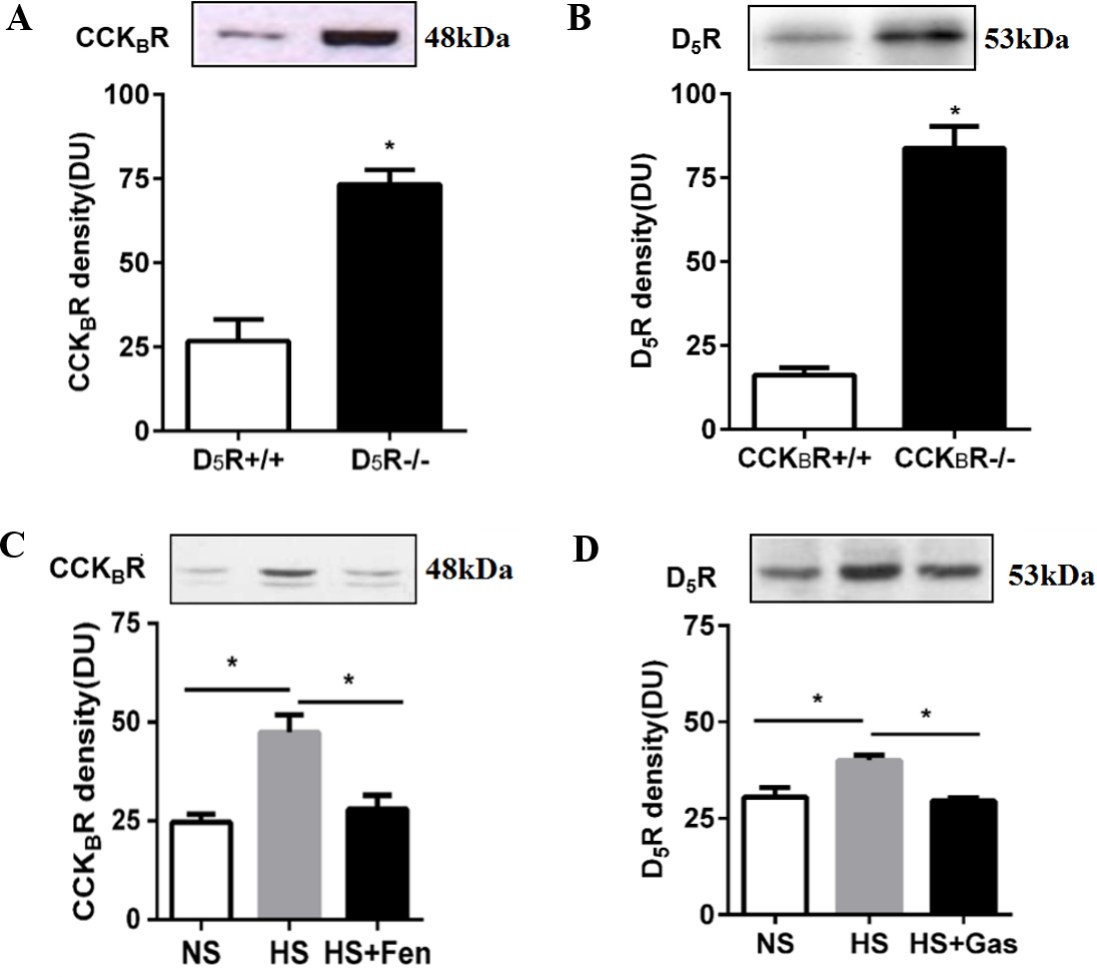
D_5_R and CCK_B_R interaction in mouse kidney. (A) CCK_B_R protein expression in PMFs is increased in D_5_R gene knockout mice (D_5_R^-/-^), relative to wild-type littermates (D_5_R^+/+^). (B) D_5_R protein expression in PMFs is increased in CCK_B_R gene knockout mice (CCK_B_R^-/-^) relative to wild-type littermates (CCK_B_R^+/+^). (C) Effect of the D_1_R and D_5_R agonist fenoldopam (Fen, 1mg/kg/day, one week) on the renal membrane protein expression of CCK_B_R in BALB/c mice fed high salt (HS) diet. NS = normal salt. (D) Effect of gastrin (Gas, 10g/kg/day, one week) on the renal membrane protein expression of D_5_R in BALB/c mice on high salt (HS) diet. NS = normal salt. *P<0.05, n = 3–5 per group, Student’s t test. All immunoblotting results are expressed as relative density units (DU). Sample loading amount was quantified by bicinchoninic acid assay. Immunoblots of D_5_R and CCK_B_R are shown in the inset.

To test whether or not D_5_R and CCK_B_R can regulate each other’s expression *in vivo*, we measured, by immunoblotting, CCK_B_R protein expression in PMFs of D_5_R^-/-^ mice and D_5_R protein expression in PMFs of CCK_B_R^-/-^ mice. As shown in Fig 6A and 6B, CCK_B_R protein expression in PMFs was greater in D_5_R^-/-^ mice than D_5_R^+/+^ littermates (73.3±4.3 vs 26.7±6.4; n = 3–4; P<0.05); D_5_R protein expression in PMFs was also greater in CCK_B_R^-/-^ mice than CCK_B_R^+/+^ littermates (83.8±6.5 vs 16.2±2.2; n = 3–4; P<0.05).

We next assessed the interaction between renal CCK_B_R and D_5_R in BALB/c mice fed normal and high salt diets. High salt diet, relative to normal salt diet, increased both CCK_B_R protein expression in PMFs (47.4±4.4 vs 24.6±2.0; n = 3; P<0.05) (Fig 6C) and D_5_R protein expression in PMFs (40.0±1.3 vs 30.4±2.5; n = 3; P<0.05) (Fig 6D). Chronic stimulation of D_1_-like receptors by the intraperitoneal injection of fenoldopam, significantly decreased CCK_B_R protein expression in PMFs of BALB/c mice on high salt diet (27.9±3.6 vs 47.4±4.4; n = 3; P<0.05) (Fig 6C). Similarly, chronic stimulation of CCK_B_R, by the intraperitoneal injection of gastrin, also decreased D_5_R protein expression in PMFs of BALB/c mice on high salt diet (29.5±0.8 vs 40.0±1.3; n = 3; P<0.05) (Fig 6D).

### Natriuretic effect of CCK_B_R^-/-^ mice

**Fig 7:**
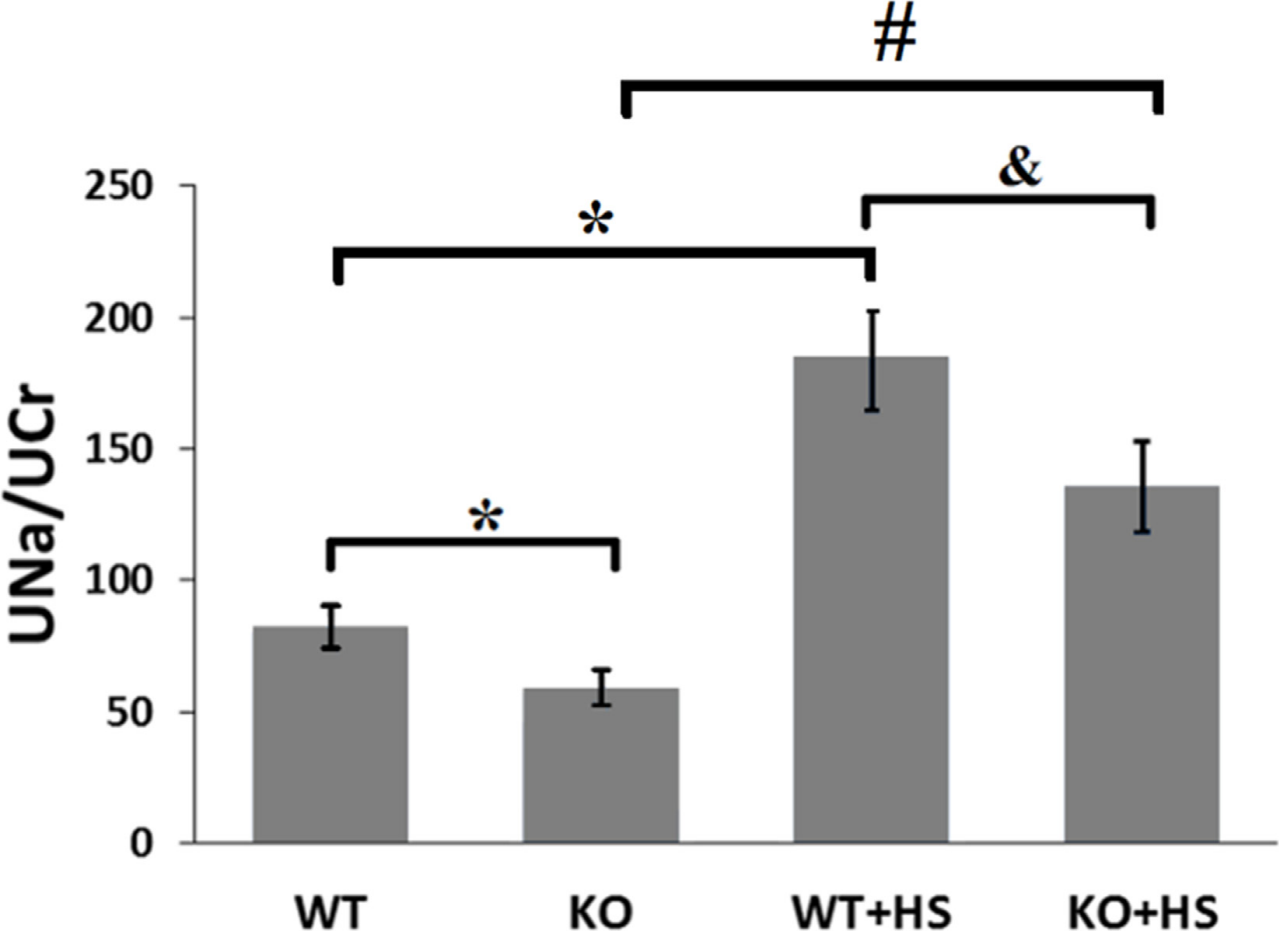
Natriuretic effect of CCK_B_R^-/-^ mice. 24-hour urine sodium excretion (UNa, mmol/L) was corrected for 24-hour creatinine excretion (UCr, mmol/L). WT = CCK_B_R^+/+^ littermates on normal salt diet (n = 8); KO = CCK_B_R^-/-^ mice on normal salt diet (n = 12); WT+HS = CCK_B_R^+/+^ littermates on high salt diet (n = 8); KO+HS = CCK_B_R^-/-^ mice on high salt diet (n = 8). *P<0.05 vs WT, &P<0.05 vs WT+HS, #P<0.05 vs KO, one-way factorial ANOVA.

D_5_R has been extensively reported as a natriuretic receptor [8,10,12,14,23,40]. In order to further explore the role of CCK_B_R in mediating renal sodium excretion, we studied the natriuretic effect of CCK_B_R^-/-^ mice and their littermates (Fig 7). CCK_B_R^-/-^ mice, in comparison with their littermates, had a notable decrease in natriuresis either on normal salt diet (59.1±6.6 vs 82.4±8.0) or high salt diet (135.7±17.0 vs 184.6±19.5).

### D_5_R and CCK_B_R interaction and natriuresis

**Fig 8:**
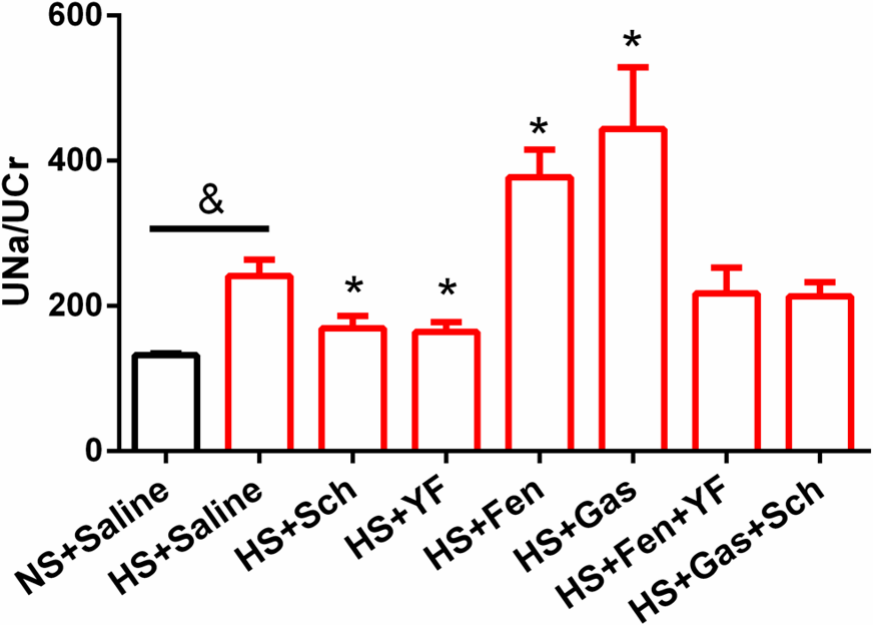
D_5_R and CCK_B_R interaction and natriuresis. 24-hour urinary sodium to creatinine ratio (UNa/UCr) was used to evaluate natriuresis. Red bars represent the groups fed high salt (HS) diet. Black bar represents the normal salt (NS) diet group. Sch = Sch23390 (D_1_R and D_5_R antagonist), Fen = Fenoldopam (D_1_R and D_5_R agonist), Gas = Gastrin, and YF = YF476 (CCK_B_R antagonist). n = 5–7, &P<0.05 vs NS, Student’s *t* test; *P<0.05 vs HS, one-way factorial ANOVA.

To determine if there is role of the interaction between D_5_R and CCK_B_R on their expression in modulating renal sodium transport, the natriuretic effect of the D_1_R and D_5_R agonist fenoldopam and gastrin in the presence or absence of their antagonists was investigated in BALB/c mice fed a high salt **(**3% NaCl) diet. As shown in Fig 8, the urine sodium excretion was significantly increased (241.4±22.4 vs 132.1±2.5; n = 7; P<0.05) after the high salt diet. Intraperitoneal administration of the D_1_R/D_5_R antagonist Sch23390 or the CCK_B_R antagonist YF476 evidently decreased the sodium excretion (169.4±16.8 vs 241.4±22.4 and 164.5±13.4 vs 241.4±22.4; n = 7; P<0.05), compared with the group of high salt diet. Fenoldopam significantly increased the natriuresis (377.4±38.0 vs 241.4±22.4; n = 6–7; P<0.05) in the mice fed high salt diet that was blocked by the CCK_B_R antagonist YF476. Similarly, gastrin evidently increased the natriuresis (443.7.4±85.3 vs 241.4±22.4; n = 6–7; P<0.05) in the mice fed high salt diet that was also blocked by the D_1_R/D_5_R antagonist, Sch23390. These results demonstrate that CCK_B_R and D_5_R interact with each other in increasing the natriuresis caused by their respective agonists in mice fed high salt diet.

## Discussion

Cross-transplantation studies between hypertensive and normotensive strains of rats and mice have provided convincing evidence for the role of the kidney in the regulation of blood pressure [40–42]. The kidney is the paramount organ in the regulation of sodium balance, an impairment of which causes a shift of the pressure-natriuresis curve “to the right”; a higher blood pressure is needed to excrete the same amount of sodium [43]. Relative to the other nephron segments, the RPT is responsible for reabsorption of >65% of filtered salt and water [42,44]. The renal regulation of sodium balance involves a cross-talk between natriuretic or anti-natriuretic factors acting on RPT, exemplified by the dopaminergic and renin-angiotensin (AT_1_R and AT_2_R) systems [6,8–14,23,33,35,40,42,44–48].

Our previous study, in agreement with other studies, verified that another natriuretic hormone, gastrin, from the gastrointestinal tract, may act in the kidney, including the RPT, to inhibit sodium transport [14,18–20]. Our results demonstrate that knockout of gastrin receptor (CCK_B_R) gene in mice results in high blood pressure that can be aggravated in response to an oral salt load, which is in accordance with an early study [18]. Gastrin can inhibit Na^+^-K^+^-ATPase activity in intestinal mucosa [49] and RPT cells [14]. Gastrin may increase the level of cAMP and the activities of some signal transducers, for example, protein kinase A and C (PKA and PKC), to cause a decrease in Na^+^-K^+^-ATPase activity, directly or indirectly [19,50–53]. Gastrin has been reported to increase NHE activity in pancreatic acini [54,55] but this is due mainly to NHE1 [56], although NHE3 is expressed in pancreatic duct cells [57]. By contrast, we have reported that gastrin inhibits NHE3 activity in human RPT cells by a phosphoinositide 3-kinase-/PKC dependent pathway [19] and Na^+^-K^+^-ATPase activity in rat RPT cells [14]. To test the possibility that the high blood pressure of CCK_B_R^-/-^ mice is related to a decreased ability to excrete a sodium load that elicited by the inhibition of sodium handling, sodium excretion studies in CCK_B_R^-/-^ mice and their littermates were performed. Our results show that CCK_B_R^-/-^ mice excrete less sodium than CCK_B_R^+/+^ littermates either on normal salt diet or high salt diet.

Our previous study demonstrated a synergistic interaction between gastrin and renal dopamine, presumably acting at the D_1_R, in increasing renal sodium excretion [14]. Dopamine produced by the RPT, independent of renal nerves and not converted to norepinephrine, is important in facilitating the excretion of sodium after a moderate sodium load [8–14,40,42,44–48,58]. Prevention of the RPT production of dopamine [59] or deletion of any of the dopamine receptor subtypes [10] results in hypertension that is dopamine receptor subtype specific. Dopamine, via all its receptors, decreases renal sodium transport by inhibiting the activity of sodium exchangers, channels, and pump [8–14,23,42,44,46–48,58–60]. In the present study, we found a concentration- and time-dependent synergistic interaction between CCK_B_R and the other dopamine D_1_-like receptor, D_5_R. Because the over expression of proteins can result in promiscuous associations, we first used HK-2 and human RPT cells that endogenously express D_5_R and CCK_B_R. Although HK-2 cells retain many functional characteristics of RPTCs, a study discovered that HK-2 cells are uncoupled from D_1_R adenylyl cyclase stimulation [61]. Therefore, we verified this effect in NT cells obtained from a normotensive white male (S2 Fig); the D_1_-like receptor agonist fenoldopam-stimulated cAMP accumulation was similar in HK-2 and NT cells (S2 File and S3 Fig). These results demonstrated that our HK-2 cells have normal D_1_-like receptor adenylyl cyclase coupling, in agreement with other reports [60,62]. Although there is no agonist that is selective to D_1_R or D_5_R [9,10,12–14], we used a specific D_5_R antagonist, LE-PM436 [23,33] to verify the involvement of the D_5_R in our studies (S4 Fig). In order to rule out any confounding effect of the D_1_R, we co-expressed human D_5_R and CCK_B_R in HEK-293 cells (HEK293-D_5_R-CCK_B_R). In HEK293-D_5_R-CCK_B_R cells, we found that D_5_R and CCK_B_R regulated each other’s total cellular expression at both the protein and mRNA levels, suggesting that the regulation may occur at the transcriptional level.

We next tested if the D_5_R and CCK_B_R interaction *in vitro* has significance *in vivo*. We found that a high salt diet increased both D_5_R and CCK_B_R proteins expression in PMFs. However, in contrast to the ability of either receptor to increase each other’s total expression in cells *in vitro*, we found that the stimulation of one receptor actually decreased the other receptor protein expression in PMFs of sodium-loaded BALB/c mice. We did not study the mechanism of this finding. However, the stimulation of membrane bound receptors should result in their internalization [63], thus the decrease in D_5_R and CCK_B_R expressions in PMFs with gastrin and fenoldopam treatment, respectively. This may also explain why the disruption of one receptor (e.g. D_5_R^-/-^, CCK_B_R^-/-^) increased the other receptor expression in PMFs, i.e., there is no physical interaction and therefore, no internalization. Because D_5_R and CCK_B_R are both natriuretic receptors, disruption of either receptor may cause a compensatory increase in the protein expression of the other. In other words, short-term stimulation of one receptor may result in transient activation or increased expression of the other but continuous stimulation should result in desensitization. Because D_5_R and CCK_B_R physically interact, the desensitization of one may desensitize the other by their internalization or even decreased expression, if they are routed to proteasomes or lysosomes. We have reported that D_5_R^-/-^ mice have increased sympathetic tone [64], which can result in vagal inhibition, releasing the slow inhibitory effect on gastrin release [65]. Therefore, we suggest that *in vivo*, intricate neural and hormonal mechanisms may participate in the mutual D_5_R and CCK_B_R regulation. However, the specific mechanism remains to be further explored.

Acute renal perfusion experiments have showed the synergistic effect of D_1_-like receptors, presumably D_1_R, and CCK_B_R on natriuresis [14]. In this study, we used the 24-hour urinary sodium to creatinine ratio [66] to evaluate the long-term effect of salt intake, gastrin, fenoldopam, and their respective receptor antagonists on urinary sodium excretion. High salt diet significantly increased water and food intake in BALB/c mice (S3 Table), that agrees with a commonly held belief that salt intake arouses thirst and increases food consumption [67,68]. High salt diet induced an augment in urinary sodium concentration. We think this, in part, may be an indirect consequence of activation of natriuretic receptors, including D_5_R and CCK_B_R. Two weeks of high salt diet had no effect on the body weights and blood pressures (S3 Table) of BALB/c mice that are salt resistant. But, to be in balance, high salt groups may excrete a larger urine volume than normal salt diet. Intraperitoneal administration of the D_1_R/D_5_R agonist fenoldopam promoted a natriuresis in salt-loaded BALB/c mice, which was not observed in D_5_R^-/-^ mice which are hypertensive [27,40,64,69]. The presence of intact D_1_R in D_5_R^-/-^ mice does not make any difference probably because the D_1_R and D_5_R physically interact in the inhibition of renal sodium transport [23,33]. D_5_R^-/-^ mice, on normal and high sodium diet have increased renal expression of sodium co-transporters, NKCC2 and NCC, and γ subunits of ENaC; on high salt diet renal NHE3 expression was also increased [69]. The increased renal expression of sodium co-transporters and channels may be responsible for the impaired ability of D_5_R-/- mice to maintain a normal sodium balance, shifting the pressure-natriuresis plot to the right [26,27,69]. In our results, fenoldopam enhanced the natriuresis in sodium loaded BALB/c mice, which was abrogated by YF476, a potent and selective CCK_B_R antagonist while the increase in natriuresis caused by gastrin was abolished by Sch23390, a D_1_R and D_5_R antagonist. The pharmacological assays have no significant effect on water intake, food intake and blood pressure (S3 Table), indicating that the variation of urinary sodium to creatinine ratio is not the results of different sodium consumption. Both fenoldopam [70] and gastrin [71] have been previously confirmed to have no effect on food intake, which can further support our results.

In summary, we have demonstrated that CCK_B_R and D_5_R synergistically interact in the kidney. The cooperative effect of D_5_R and CCK_B_R may be involved in the maintenance of normal sodium and water balance when salt intake is increased.

## Supporting Information

S1 Fig HEK293-D_5_R-CCK_B_R cells heterologously overexpressing human D_5_R and CCK_B_R. D_5_R and CCK_B_R mRNA (A and B) and protein (C) expressions in co-transfected HEK293-D_5_R-CCK_B_R cells. HDC: HEK293-D_5_R-CCK_B_R cell total mRNA; NC: negative control, HEK293 cell total mRNA; Blank: H_2_O; PC: positive control, plasmid including the human D_5_R or CCK_B_R gene. GAPDH (36kDa) is used for the correction of protein loading.
(TIF)

S2 Fig D_5_R and CCK_B_R co-regulation in NT cells. (A) Effects of fenoldopam (10^−6^ mol/L, 24 hours) and D_1_R/D_5_R antagonist Sch23390 (10^-6^mol/L, 24 hours) on CCK_B_R protein expression (n = 6, *P<0.05 vs control, one-way factorial ANOVA, Duncan’s test). (B) Effects of gastrin (10^-8^mol/L, 24 hours) and CCK_B_R antagonist YF476 (10^-8^mol/L, 24 hours) on D_5_R protein expression (n = 6, *P<0.05 vs control, one-way factorial ANOVA, Duncan’s test). All immunoblotting results are expressed as relative density units (DU) and normalized by GAPDH expression. Immunoblots of D_5_R, CCK_B_R, and GAPDH are shown in the inset.
(TIF)

S3 Fig Detection of fenoldopam-stimulated cAMP accumulation in both HK-2 and NT cells. White bar represents HK-2 cells; black bar represents NT cells. cAMP production is expressed as nanogram (ng) per liter of solution (n = 6, *P<0.05 vs others, one-way factorial ANOVA, Duncan’s test).
(TIF)

S4 Fig Verification of the specificity of the interaction between D_5_R and CCK_B_R using a selective D_5_R antagonist, LE-PM436. (A) HK-2 cells, (B) NT cells. Effects of fenoldopam (10^−6^ mol/L, 24 hours) and D_5_R antagonist LE-PM436 (10^-6^mol/L, 24 hours) on CCK_B_R protein expression (n = 5, *P<0.05 vs control, one-way factorial ANOVA, Duncan’s test). All immunoblotting results are expressed as relative density units (DU) and normalized by GAPDH expression. Immunoblots of CCK_B_R and GAPDH are shown in the inset.
(TIF)

S1 File Plasma membrane-enriched fractions (PMFs) extraction. (DOCX)

S2 File Determination of cAMP accumulation. (DOCX)

S1 Table Chemical drugs, antibodies, and test kits. (DOCX)

S2 Table Primers used for RT-PCR or qRT-PCR. (DOCX)

S3 Table Water intake, food intake, body weight and MAP of BALB/c mice. (DOCX)
