# Supplementary figures and images for: The Synergistic Roles of Cholecystokinin B and Dopamine D5 Receptors on the Regulation of Renal Sodium Excretion
Source: PLoS One. 2016 Jan 11;11(1):e0146641. doi: 10.1371/journal.pone.0146641 (PMC4709046; doi:10.1371/journal.pone.0146641)

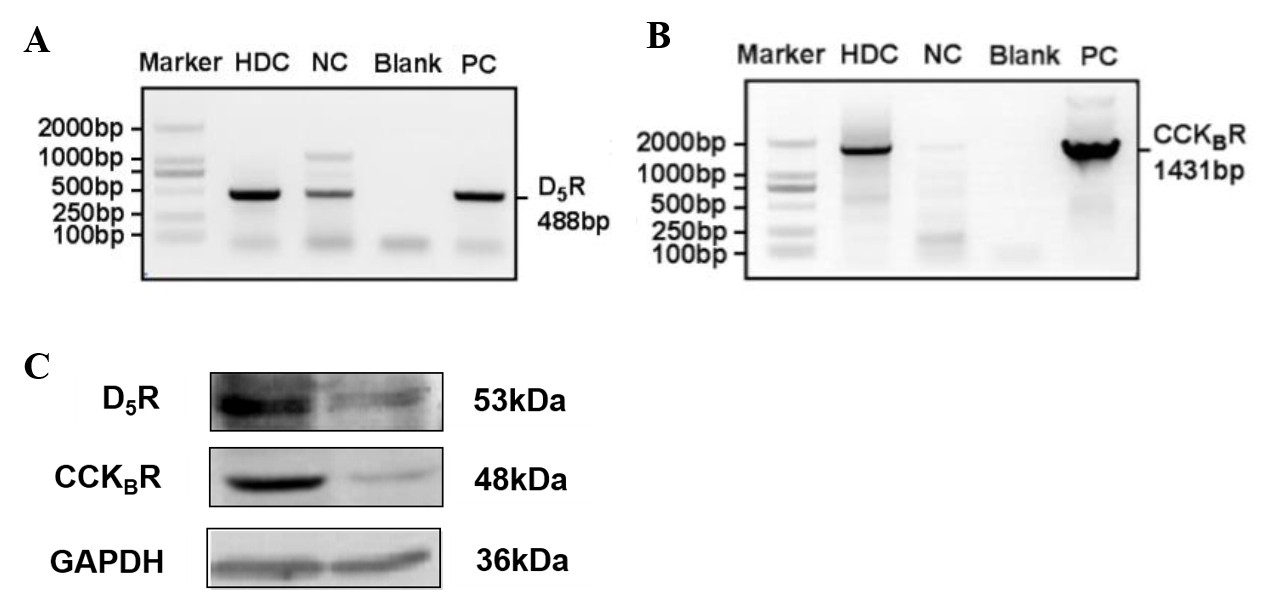

Supplement: S1 Fig — D5R and CCKBR mRNA (A and B) and protein (C) expressions in co-transfected HEK293-D5R-CCKBR cells. HDC: HEK293-D5R-CCKBR cell total mRNA; NC: negative control, HEK293 cell total mRNA; Blank: H2O; PC: positive control, plasmid including the human D5R or CCKBR gene. GAPDH (36kDa) is used for the correction of protein loading. (TIF) [file pone.0146641.s001.tif]

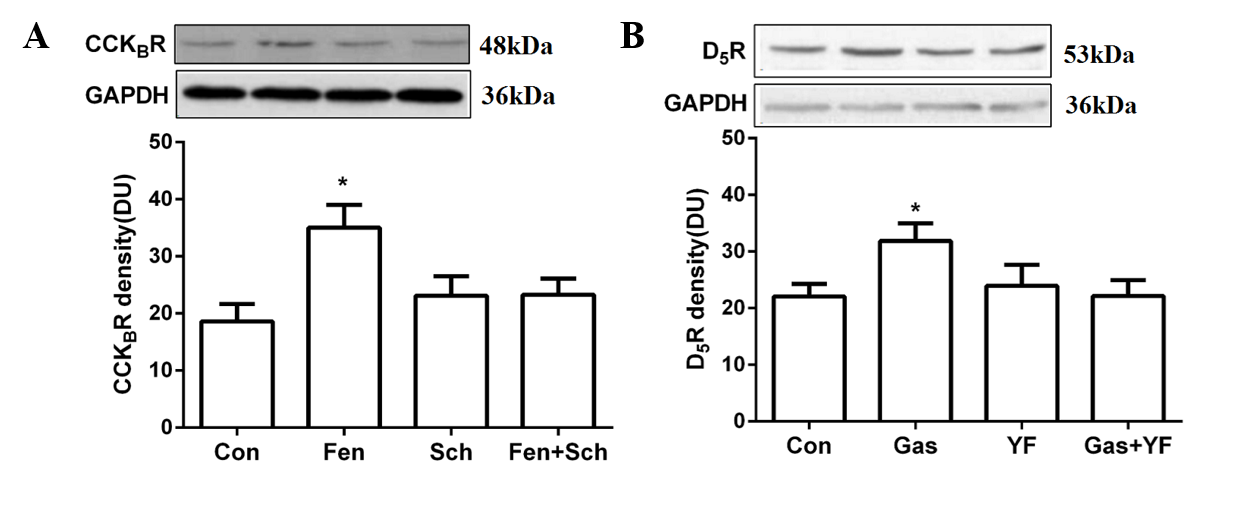

Supplement: S2 Fig — (A) Effects of fenoldopam (10−6 mol/L, 24 hours) and D1R/D5R antagonist Sch23390 (10-6mol/L, 24 hours) on CCKBR protein expression (n = 6, *P<0.05 vs control, one-way factorial ANOVA, Duncan’s test). (B) Effects of gastrin (10-8mol/L, 24 hours) and CCKBR antagonist YF476 (10-8mol/L, 24 hours) on D5R protein expression (n = 6, *P<0.05 vs control, one-way factorial ANOVA, Duncan’s test). All immunoblotting results are expressed as relative density units (DU) and normalized by GAPDH expression. Immunoblots of D5R, CCKBR, and GAPDH are shown in the inset. (TIF) [file pone.0146641.s002.tif]

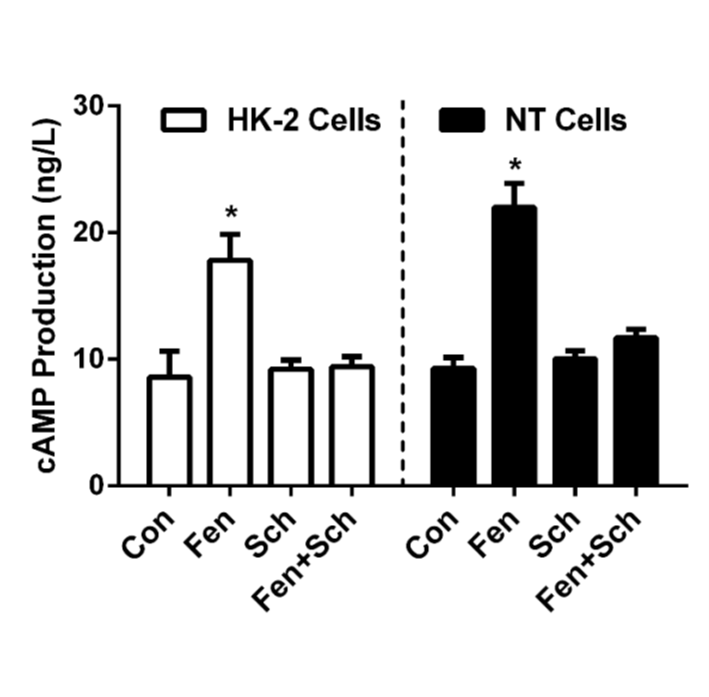

Supplement: S3 Fig — White bar represents HK-2 cells; black bar represents NT cells. cAMP production is expressed as nanogram (ng) per liter of solution (n = 6, *P<0.05 vs others, one-way factorial ANOVA, Duncan’s test). (TIF) [file pone.0146641.s003.tif]

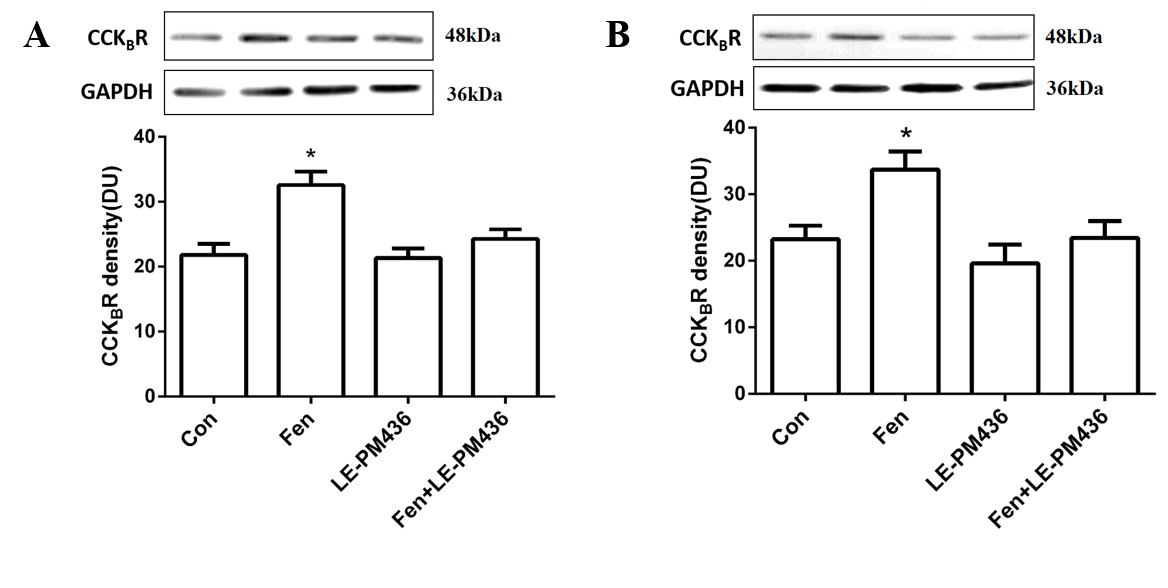

Supplement: S4 Fig — (A) HK-2 cells, (B) NT cells. Effects of fenoldopam (10−6 mol/L, 24 hours) and D5R antagonist LE-PM436 (10-6mol/L, 24 hours) on CCKBR protein expression (n = 5, *P<0.05 vs control, one-way factorial ANOVA, Duncan’s test). All immunoblotting results are expressed as relative density units (DU) and normalized by GAPDH expression. Immunoblots of CCKBR and GAPDH are shown in the inset. (TIF) [file pone.0146641.s004.tif]
