# Supplementary material for: The Synergistic Roles of Cholecystokinin B and Dopamine D5 Receptors on the Regulation of Renal Sodium Excretion
Source: PLoS One. 2016 Jan 11;11(1):e0146641. doi: 10.1371/journal.pone.0146641 (PMC4709046; doi:10.1371/journal.pone.0146641)
Supplement: S1 File — (DOCX) [file pone.0146641.s005.docx]

**S1 File. Plasma membrane-enriched fractions (PMFs) extraction.**

PMFs were extracted using a Membrane Protein Extraction Kit (C500049, Sango Biotech, China), according to the manufacturer’s instruction. Detailed steps are described as follows. Renal cortex (200mg) was cut into small pieces on ice, washed thrice by 1 vol of pre-cooling scrubbing solution, added 1 ml extraction buffer coupled with 1 μl protease inhibitor and 1μl dithiothreitol (DTT), moved to a glass homogenizer for homogenizing and then sonically disrupted. The mixture was kept on ice for 30 minutes and briefly vortexed every 10 minutes. The homogenate was then centrifuged at 4 ℃ with 17500 g for 10 minutes. The supernatant lysates were harvested and incubated at 37 ℃ for 10 minutes and centrifuged at room temperature with 16000 g for 5 minutes. After centrifugation, the supernatant lysates were divided into two layers and the sublayer was retained and diluted in 500 μl pre-cooling three distilled water and kept at 4 ℃ for 5 minutes. The above two steps were repeated three times and the sublayer containing PMFs was retained for use. Protein concentration was determined by bicinchoninic acid assay.
