# Supplementary material for: The Synergistic Roles of Cholecystokinin B and Dopamine D5 Receptors on the Regulation of Renal Sodium Excretion
Source: PLoS One. 2016 Jan 11;11(1):e0146641. doi: 10.1371/journal.pone.0146641 (PMC4709046; doi:10.1371/journal.pone.0146641)
Supplement: S2 File — (DOCX) [file pone.0146641.s006.docx]

**S2 File. Determination of cAMP accumulation.**

HK-2 and NT cells were grown to 80% confluence, serum-starved overnight, and then washed in PBS with Ca^2+^ and Mg^2+^. Isobutyl-1-methyl-xanthine (IBMX, 10^-3^ M) was added along with a D_1_R and D_5_R agonist (fenoldopam, 10^-6^ M) or vehicle control (DMSO) and incubated for 30 minutes at 37℃. In order to show D_1_-like receptor specificity of the measured end points, D_1_/D_5_ receptor antagonist (Sch23390, 10^-5^ M) was added 10 minutes prior to the addition of fenoldopam. Human cAMP ELISA kit was performed according to manufacturer’s specifications.
