# Supplementary material for: The Synergistic Roles of Cholecystokinin B and Dopamine D5 Receptors on the Regulation of Renal Sodium Excretion
Source: PLoS One. 2016 Jan 11;11(1):e0146641. doi: 10.1371/journal.pone.0146641 (PMC4709046; doi:10.1371/journal.pone.0146641)
Supplement: S1 Table — (DOCX) [file pone.0146641.s007.docx]

**S1 Table. Chemical drugs, antibodies, and test kits.**

| Name | Catalog Number | Manufacturer | Country |
| --- | --- | --- | --- |
| DMEM/F12 | M1805 | Bioroc | China |
| Fetal bovine serum | 16000-044 | Gibco | USA |
| pCMV6-AC-CCK_B_R plasmid | SC319418 | OriGene | USA |
| RIPA lysis buffer | P0013 | Beyotime | China |
| Protein A/G | sc-2003 | Santa Cruz | USA |
| Trizol | 15596-026 | Invitrogen | USA |
| Lipofectamine 2000 transfection reagents | 12566-014 | Invitrogen | USA |
| Membrane Protein Extraction Kit | C500049 | Sango Biotech | China |
| SuperScript III | 11732-088 | Invitrogen | USA |
| One Step SYBR PrimeScript RT-PCR Kit | RR066A | Takara | Japan |
| Human cAMP ELISA kit | XF00006B | Xinfan | China |
| Bicinchoninic acid | P0011 | Beyotime | China |
| Mouse polyclonal anti-D_5_R antibody | sc-33661 | Santa Cruz | USA |
| Rabbit polyclonal anti-CCK_B_R antibody | NBP1-00744 | NOVUS | USA |
| GAPDH | KC5G5 | KangChen | China |
| Mouse polyclonal anti-CCK_B_R antibody | sc-166690 | Santa Cruz | USA |
| Rabbit polyclonal anti-D_5_R antibody | [sc-25650](http://www.scbt.com/search/redirect.php?location=datasheet-25650-d5dr-h-107-antibody.html&searchPhrase=drd5&datasheet=sc-25650&tableName=&productType=&page=1) | Santa Cruz | USA |
| Alexa Fluor 568-labeled goat anti-rabbit IgG | ab175471 | Abcam | USA |
| HRP-conjugated goat anti-mouse IgG | ZDR-5306 | ZSGB-BIO | China |
| HRP-conjugated goat anti-rabbit IgG | ZB-2301 | ZSGB-BIO | China |
| Alexa Fluor 488-labeled goat anti-mouse IgG | ab150113 | Abcam | USA |
| Penicillin-Streptomycin | 15140-148 | Gibco | USA |
| Neomycin | N6386 | Sigma | USA |
| Fenoldopam | SML0198 | Sigma | USA |
| Gastrin | RP12740 | Genscript | USA |
| Sch23390 | D054 | Sigma | USA |
| Isobutyl-1-methyl-xanthine | 28822-58-4 | Mp | USA |
| YF476 | unknown unknown | | unknown |
| LE-PM436 | unknown unknown | | unknown |

Note: LE-PM436 was a gift from Dr. Christoph Enzensperger.
