# Supplementary material for: The Synergistic Roles of Cholecystokinin B and Dopamine D5 Receptors on the Regulation of Renal Sodium Excretion
Source: PLoS One. 2016 Jan 11;11(1):e0146641. doi: 10.1371/journal.pone.0146641 (PMC4709046; doi:10.1371/journal.pone.0146641)
Supplement: S2 Table — (DOCX) [file pone.0146641.s008.docx]

**S2 Table. Primers used for RT-PCR or qRT-PCR.**

|  | **Forward primer** | **Reverse primer** |
| --- | --- | --- |
| D_5_R | 5'-GGACCGCTACTGGGCCATCT-3' | 5'-GGGTCTTGAGAACCTTGGTC-3' |
| CCK_B_R | 5'-GATGAGCGTTGGAGGAAAT-3' | 5'-CAGCCTGTTGGTCAGAGGTATG-3' |
| D_5_R | 5'-ATCATGTGCTCCACTGCCTC-3' | 5'-CCATGCGCTGAGTCATCTTG-3' |
| CCK_B_R | 5'-CCTACGGGCTTATCTCTCGC-3' | 5'-TTCTGGTGAACAGCCCCTGG-3' |
| GAPDH | 5'-CTCCACCTTTGACGC-3' | 5'-CCACCCTGTTGCTGT-3' |

Note: the first two are for RT-PCR; the rest are for qRT-PCR.
