# Supplementary material for: The Synergistic Roles of Cholecystokinin B and Dopamine D5 Receptors on the Regulation of Renal Sodium Excretion
Source: PLoS One. 2016 Jan 11;11(1):e0146641. doi: 10.1371/journal.pone.0146641 (PMC4709046; doi:10.1371/journal.pone.0146641)
Supplement: S3 Table — (DOCX) [file pone.0146641.s009.docx]

**S3 Table. Water intake, food intake, body weight and MAP of BALB/c mice.**

|  | **Water**  **(ml/day)** | **Food**  **(g/day)** | **Body Weight**  **(g)** | **MAP**  **(mmHg)** |
| --- | --- | --- | --- | --- |
| NS | 3.2±0.1 | 3.4±0.1 | 23.4±0.4 | 77.0±3.7 |
| HS | 5.2±0.1* | 4.5±0.2* | 23.7±0.7 | 79.3±3.0 |
| HS+Sch | 4.9±0.3 | 4.5±0.4 | 22.5±0.6 | 83.3±4.0 |
| HS+YF476 | 4.8±0.3 | 4.4±0.4 | 24.4±0.5 | 81.0±4.7 |
| HS+Fen | 5.0±0.2 | 4.3±0.4 | 22.5±1.0 | 77.3±3.3 |
| HS+Gas | 5.0±0.2 | 4.4±0.3 | 23.6±0.5 | 76.3±5.1 |
| HS+Fen+YF476 | 4.4±0.3 | 4.3±0.2 | 21.6±0.9 | 75.6±1.6 |
| HS+Gas+Sch | 4.8±0.3 4.2±0.2 | | 22.6±0.7 | 80.0±4.1 |

Note: *P<0.05 vs NS, one-way factorial ANOVA.
